# The clinical effects of modified tinnitus relieving sound (MTRS) for chronic tinnitus: protocol for a randomized controlled trial

**DOI:** 10.1186/s13063-023-07389-8

**Published:** 2023-06-02

**Authors:** Dongmei Tang, Jinghan Wang, Xiaopei Yu, Huiqian Yu

**Affiliations:** 1 ENT Institute and Otorhinolaryngology Department of Eye & ENT Hospital, State Key Laboratory of Medical Neurobiology and MOE Frontiers Center for Brain Science, Fudan University, Shanghai, 200031 People’s Republic of China; 2 NHC Key Laboratory of Hearing Medicine, Fudan University, Shanghai, 200031 People’s Republic of China

**Keywords:** Tinnitus, Acoustic therapy, Modified tinnitus relieving sound (MTRS), Unmodified music (UM)

## Abstract

**Introduction:**

Chronic subjective tinnitus has become an increasingly serious hazard that affects the health-related quality of life for millions of people. Due to the lack of curative treatment strategies, this study aims to introduce a novel acoustic therapy named the modified tinnitus relieving sound (MTRS) for tinnitus and to evaluate the efficacy of MTRS in comparison with unmodified music (UM) which served as a control.

**Methods and analysis:**

A randomized, double-blinded, controlled, clinical trial will be carried out. Sixty-eight patients with subjective tinnitus will be recruited and randomly allocated into two groups in 1:1 ratio. The primary outcome is Tinnitus Handicapped Inventory (THI); the secondary outcomes are the Hospital Anxiety and Distress Scale (HADS; HADS subscales for Anxiety (HADS-A) and Depression (HADS-D)), Athens Insomnia Scale (AIS), the visual analog scale (VAS) for tinnitus, and tinnitus loudness matched by sensation level (SL). Assessment will be performed at baseline and at 1, 3, 9, and 12 months post-randomization. The sound stimulus will be persistent until 9 months after randomization, and be interdictory in the last three months. Data collected during the intervention process will be analyzed and compared to baseline.

**Ethics and dissemination:**

This trial received ethical approval from the Institutional Review Board (IRB) of Eye & ENT Hospital of Fudan University (No. 2017048). The study results will be disseminated via academic journals and conferences.

**Funding:**

This study is supported by the Shanghai Shenkang Development Program (SHDC12019119), the Excellent Doctors-Excellent Clinical Researchers Program (SYB202008), the Shanghai Rising-Star Program (23QC1401200), the Shanghai Rising Stars of Medical Talent Youth Development Program (2021–99), the National Natural Science Foundation of China (81800912), and the National Natural Science Foundation of Shanghai (21ZR1411800).

**Trial registration:**

ClinicalTrials.gov NCT04026932. Registered on 18 July 2019.

## Strengths and limitations of this study

- The modified tinnitus relieving sound (MTRS) has a variable frequency, time, and intensity domains, which is more acceptable to patients and promotes relief from negative emotions (e.g., irritability, anxiety, and depression).
- The integrated MTRS and otologist-guided cognitive behavioral therapy for tinnitus (CBT-T) may be a more effective strategy for tinnitus sufferers.
- The randomized controlled study allows for high-quality evidence-based clinical trials for tinnitus treatment using modulated sound.
- Acute subjective tinnitus and objective tinnitus sufferers, as well as participants who cannot communicate in Mandarin, are excluded, thus the recruitment does not cover the whole tinnitus population.

## Introduction

Tinnitus is an auditory perception of sound in the absence of a corresponding external acoustic stimulus [1], which has a global prevalence of approximately 10–25% among the adult population [2, 3]. The reported prevalence in children and adolescents was estimated at 6–41.9% when both hearing loss and normal hearing were included [4]. Longstanding chronic tinnitus (> 3 months) often induces negative emotions, including anxiety and depression [5, 6]. Psychologically, insomnia or sleep impairments are highly prevalent in patients with tinnitus, and the most severe sleep disorder is often associated with anxiety plus depression [7].

Tinnitus might develop as a consequence of changes that occur in central auditory pathways and other brain regions when the brain loses its input from the ear [8]. The non-auditory cortex network, such as the limbic system, emotional center, “salience” network, and memory center, also extensively participate in the generation and progression of tinnitus [9], as well as in the reinforcement of the associated distress, anxiety, and depression, which in turn aggravate tinnitus, forming a vicious cycle [9, 10]. Therefore, the timely and presice intervention is advocated in tinnitus treatment, whereby such a vicious cycle and tinnitus progress could be terminated in due course. However, there is currently no effective cure for tinnitus.

Present therapeutics mainly concentrate on relieving various complications induced by tinnitus, for example, depression and insomnia. Thus, education counseling, tinnitus retraining therapy (TRT), and cognitive behavioral therapy (CBT) are frequently adopted in tinnitus treatment [11]. TRT combines tinnitus-specific educational counseling and low-level broadband sound therapy, aiming to facilitate habituation of the awareness of tinnitus and to reduce the negative emotion evoked by tinnitus [12, 13]. Various studies have reported the effectiveness of TRT in improving the quality of life of tinnitus patients, however, most of the studies were uncontrolled or in low-quality of evidence [14–18]. Several studies compared TRT with other interventions (e.g., tinnitus masking, counseling, or different type of sounds) or compared between total and partial masking strategies of TRT, nevertheless, they found that there had no significant difference between different conditions [19–21]. A recent randomized clinical trial reported the efficacy of 151 participants with tinnitus at 6 US military hospitals who underwent 18 months of follow-up choosing TRT with conventional sound generator, TRT with placebo sound generator, or standard of care; however, they found that there were few differences among the three treatment groups [22]. Besides, it seemed that TRT did not reduce the loudness level of tinnitus but only promoted the habituation of tinnitus according to the present reports [23]. CBT for tinnitus includes various psychological interventions (e.g., behavioral activation, mindfulness, exposure, counseling, relaxation), which are conducted face-to-face or remotely online, aiming to ameliorate tinnitus-related quality of life, rather than change the perceived loudness of tinnitus [24–26]. Evidence from a Cochrane review on CBT for tinnitus has shown that CBT may be effective in reducing the negative impact of tinnitus; however, a longer-term follow-up over 6 or 12 months is still in the absence of evidence [24].

Based on the accumulated explorations on the relevant background and our clinical experience in tinnitus diagnosis and treatment, we presume that customized acoustic therapy, coupled with tinnitus-specific psychological counseling, might be powerful for persistent tinnitus relief. Therefore, we designed a new treatment system, optimizing the education counseling and introducing a customized relieving sound therapy model that is designated as MTRS. Our method has been authorized by China National Invention patent (ZL201510165976.9). To verify the safety and clinical efficacy of MTRS, we are conducting the current randomized controlled clinical study: participants in the control group receive the unmodified music (UM) treatment, while subjects in the experimental group will be given the MTRS treatment, and all participants should accept the follow-up visit at regular intervals to supervise the tinnitus variation. Our research hypothesis is that the scores of tinnitus evaluation scales of the MTRS group are significantly lower than that of UM group, whereby our current trial could provide the corresponding clinical evidence.

## Methods and analysis

### Study design and setting

This study was designed as a randomized, double-blinded, and controlled clinical trial, lasting for 30 months. The main hypothesis was that MTRS had superiority over UM in the treatment of tinnitus. Participants will be randomized into two groups in 1:1 ratio, given parallel interventions. Participants, investigators, and analysts are blinded to group allocations.

### Patient and public involvement

Participants will be recruited from outpatient clinics of the Eye & ENT Hospital of Fudan University, which is the largest ear, nose and throat specialized hospital in China and serves millions of patients from all over the country. Investigators and analysts involved are all well-trained according to standard protocols.

### Eligibility criteria

Patients who meet all of the following inclusion criteria will be considered eligible:

1. Adults aged between 18 and 80 years old;
2. Diagnosed with subjective tinnitus;
3. Chronic tinnitus: tinnitus course ≥ 3 months;
4. Be able to understand and communicate in Mandarin;
5. The average pure tone threshold (0.5, 1, 2 kHz) ≤ 55 dB HL of the worse ear;
6. Subjects are able to understand the purpose of the study, volunteer to participate and cooperate with the instructors to complete the experiment, and be willing to sign the informed consent.

Patients with any of the following conditions will be excluded:

1. Pulsatile tinnitus and objective tinnitus;
2. Having significant health issues that affect or prevent participation or continue with the follow-up;
3. Diseases requiring other medical intervention first (e.g., infections, tumors, otosclerosis, Meniere's disease, the acute stage of sudden sensorineural hearing loss);
4. People with severe hyperacusis, severe anxiety, depression, and other psychiatric disorders;
5. Currently participating in other research projects that may affect tinnitus; and
6. Subjects who are not considered suitable for this clinical trial by the researchers.

### Randomization and allocation

The total sample size is 68, and these participants will be randomly divided into two groups, 34 each. Eligible patients will be marked by random numbers. We carry out a randomization process using SPSS 12: first, set the random seed and record, generate random numbers for each participant, and then sort the random numbers in ascending order. The first 34 are the first group (UM group), and the last 34 are the second group (MTRS group). The concealed randomization sequence file will be constructed and kept in sequentially numbered, sealed, opaque envelopes by a staff member in the Eye and ENT Hospital of Fudan University outside the study team. When a patient is officially enrolled, the staff member will be called by telephone and open the corresponding envelope to find the randomized treatment for this patient. Assessors in examination rooms and statistician analysts are not allowed to receive information of the group allocation.

### Interventions

The two treatment groups are the unmodified music (UM) group and the modified tinnitus relieving sound (MTRS) group. The former is the control group and the latter is the experimental group. Acoustic stimulation is the only intervention method. Drugs and hearing aids are not considered. Participants in UM group will listen to unmodified light music at least 2 h a day for 9 months after allocation and be interdictory to any music stimulus in the following 3 months. While participants in the MTRS group will listen to modified tinnitus relieving sound, other conditions remain the same as UM group. Our research team has designed and invented one special audio processing software (APP) to compose the MTRS.

The process and output paths of MTRS are as follows: (I) detailed pure-tone audiometry test to determine the precise audiogram; (II) tinnitus position confirmation: match the tinnitus frequency (Hz) and loudness (dB) with a series of frequencies within audiometric range, namely tinnitus pitch match (PM) and loudness match (LM); (III) verification of endpoints of modulated range: based on our particular algorithm, calculate total four endpoints adjacent to tinnitus pitch; (IV) determination of the intensified modulated frequency band: demarcate two frequency bands of 1/3 octave interval below and above the tinnitus pitch according to the endpoints calculated from procedure III; and (V) the peaceful music modulation: with the help of our patented product, the tailor-made audio processing software/APP, to modulate the selected peaceful music, dynamically enhancing the intensity of music within both frequency bands with the 10 dB gains.

When participants enrolled, well-trained specialists in tinnitus or expert clinic will introduce the listening guidance, demonstrate operating steps and conduct an otologist-leading cognitive behavioral therapy for tinnitus (CBT-T), and the main components are as follows: the interpretation of tinnitus tests results, the relationship between tinnitus and hearing loss, the causes and basic pathogenesis of tinnitus, the theoretical basis and primary process of MTRS treatment, the psychoacoustic characteristics of tinnitus, the relationship between tinnitus and attention, instructions on developing common methods to shift attention, and tinnitus relaxation exercises as well. This propaganda and education would help patients enhance their self-confidence to overcome tinnitus and to ease anxiety or other negative emotions.

### Procedure

Researchers plan to recruit volunteers in the Tinnitus Special Clinic of Eye & ENT Hospital of Fudan University. According to the corresponding criteria, eligible volunteers will be detailly introduced to the trial objective and process, and also, sign the informed consent before participation. For potential participants, the investigator will conduct the consent/assent interview face to face. Participants (a natural person that has full civil capacity) will provide written permission for their own and this process will be recorded. Formal recruitment and consent/assent interviews will be conducted in Mandarin (Chinese). Prior to allocation, baseline data will be gathered from all participants, which include (I) modularized and structured interrogations of tinnitus diagnosis: referring to region, process, tonality trait, impact degree, and correlative factor of tinnitus, as well as the personal details, such as age, gender, past medical history, and treatment history; (II) audiology tests: pure tone audiometry (PTA), acoustic immittance (AI), acoustic stapedius reflex (ASR), distortion product otoacoustic emission (DPOAE), pitch match (PM) and loudness match (LM) test of tinnitus, and residual inhibition test (RI); and (III) Multi-dimensional assessment scale of tinnitus: Tinnitus Handicap Inventory (THI), Hospital Anxiety and Distress Scale (HADS-A, HADS-D subscales), Athens Insomnia Scale (AIS), and visual analog scale (VAS).

As soon as participants are allocated, current acoustic therapy starts. Participants will be provided with unmodified music (UM) and modified tinnitus relieving sound (MTRS) respectively, according to their corresponding group number. They are required to receive the aforementioned acoustic stimulation more than 2 h per day and repeatedly complete the multi-dimensional assessment scale of tinnitus at 1, 3, 9, and 12 months after commencing treatment. The follow-up evaluation will be obtained through an appointed outpatient service, or online APP, WeChat, and phone call visit, by which the duration of acoustic stimulation will be recorded faithfully.

Before the acoustic therapy and during the follow-up visit, otolaryngologists preside at cognitive psychological counseling and tinnitus knowledge education. The contents of these counseling and education mainly include (I) interpretation of tinnitus tests results, also the relationship between tinnitus and hearing loss; (II) causes and basic pathogenesis of tinnitus; (III) theoretical basis and primary process of MTRS treatment; (IV) the psychoacoustic characteristics of tinnitus, the relationship between tinnitus and attention; (V) instructions on developing common methods to shift attention, and tinnitus relaxation exercises as well; (VI) being aware of treatment purpose, confusion and other problem of every participant, and provide an answer or available solution, also encouragement; and (VII) trying to know the exact reason of quitting trial and offer suggestions about the alternative follow-up treatment.

Any protocol or procedure amendments will need to be reviewed and approved by the IRB. Major changes to the trial will have to be approved by the sponsor and investigator. All changes to the protocols will be reflected and updated on clinicaltrials.gov.

### Withdrawal/retention of participants

Participants enrolled have the right to withdraw from the trial at any time. Participants who no longer meet the inclusion criteria or are lost follow-up will also be regarded as withdrawal. Researchers should make a detailed record of withdrawal reasons such as listening to acoustic therapy less than 1 h per day, emerging other diseases. Due to the long follow-up period and the long interval between data collection time points, participants are easily lost to follow-up, which is one of the technical difficulties of the project. In order to encourage participant retention, the study will simplify the follow-up process and waive extra examination and treatment fees. Researchers will keep in touch with participants through various methods such as phone calls, WeChat, and email, and answer their questions in time.

### Blinding

This study is double-blinded. Investigators, eligible patients, and data analysts are all blinded to the group allocations during the trial: investigators do not know which group the patients are assigned; patients do not know which treatment strategy they are given; analysts do not know which group they analyze.

### Outcome measures

#### Primary outcome

##### Tinnitus Handicap Inventory (THI)

THI is a self-assessment inventory to quantify the impact of tinnitus on everyday function. It includes 25 items to evaluate the functional, emotional, and catastrophic response reactions to tinnitus. The answer options for each question include “yes,” “sometimes,” and “no.” Answering “yes” gets 4 points, answering “sometimes” gets 2 points, and “no” gets 0 points. Add the scores of the 25 questions in the above three categories to get the THI score. The score quantifies the patient’s tinnitus disability into 5 levels: slight (0–16), mild (18–36), moderate (38–56), severe (58–76), and catastrophic (78–100). The higher the score, the more severe tinnitus is. The results of THI will not be affected by age, gender, or hearing loss, having good internal consistency reliability and construct validity.

#### Secondary outcomes

##### Hospital Anxiety and Depression Scale (HADS)

HADS is used to evaluate negative emotions related to tinnitus. HADS can be used to indicate the anxiety and depression status of clinical patients. The scale includes two subscales that are anxiety (HADS-A) and depression (HADS-D) with 7 items each, each of which is scored 0–3 points. According to the score, there are three situations: asymptomatic (0–7 points), suspicious symptoms (8–10 points), and definitely present symptoms (11–21 points), and those with a score of 8 or more are considered positive.

##### Athens Insomnia Scale (AIS)

AIS grades three categories of sleep disorder: no insomnia scoring below 4, suspicious (4–6), and insomnia (7–24).

##### Tinnitus loudness (SL)

The loudness of tinnitus is reflected in sensation level (dB SL). Sensation level is defined as the loudness value above the hearing threshold at the pitch of tinnitus.

##### Visual analog scale (VAS) for tinnitus

VAS is widely used in the clinical evaluation of symptoms, such as chronic pain. In this research, we use it to evaluate the degree of distress of tinnitus symptoms. Participants are asked to assign a 0 to 10 score according to the overall feelings of the impact of tinnitus on their own life with the help of a proper ruler. The assessment must be carried out in relation to volume and disturbance. It is easily applicable and understood by most patients. However, this is a superficial assessment, impacted by cultural, intellectual, and psychological aspects. The higher the score, the more distressed the symptoms.

### Timepoints of follow-up

There are total 5 timepoints, including the baseline when participants are allocated and 1, 3, 9, and 12 months after commencing treatment, for investigators to collect data of multi-dimensional assessment scales (THI, HADS, AIS, VAS, SL). In detail, follow-up evaluations/tests will be conducted during acoustic therapy at 1, 3, 9 months after commencing treatment, whereby the short-term and long-term treatment effects of MTRS will be comprehended. In order to verify the long-term and lasting effect of current therapy, all acoustic stimulation will be withheld from participants during 10 to 12 months after commencing treatment. Thus, the final time to gather follow-up evaluation/test data is 12 months after commencing treatment. Additionally, the data collection method, whenever during therapy, must be consistent in the way the investigator collects the baseline data at the beginning of this trial. Tinnitus match tests are proper approaches for analysts to know the frequency and loudness variations of tinnitus, which must be conducted at both the initial point (baseline data collection) and endpoint (12 months after commencing treatment). Any qualified study cases should meet these criteria: baseline data completion at the initial timepoint (T0), research data collection at 1, 3, 9, and 12 months after commencing treatment timepoints (T1, T2, T3, T4 nodes, respectively, during trial). Midway quitting and loss to follow-up will be recorded.

**Fig. 1:**
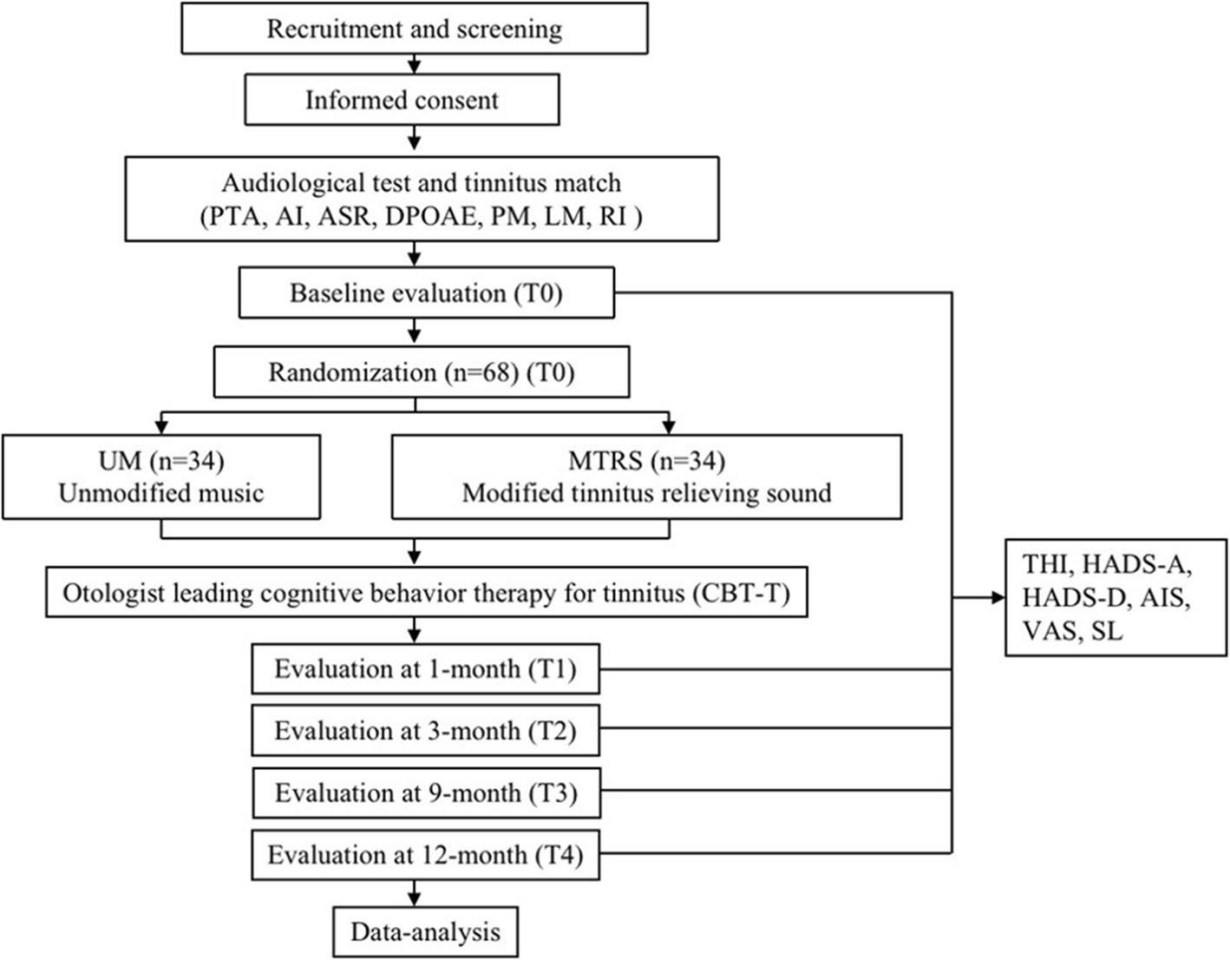
Study flowchart

**Fig. 2:**
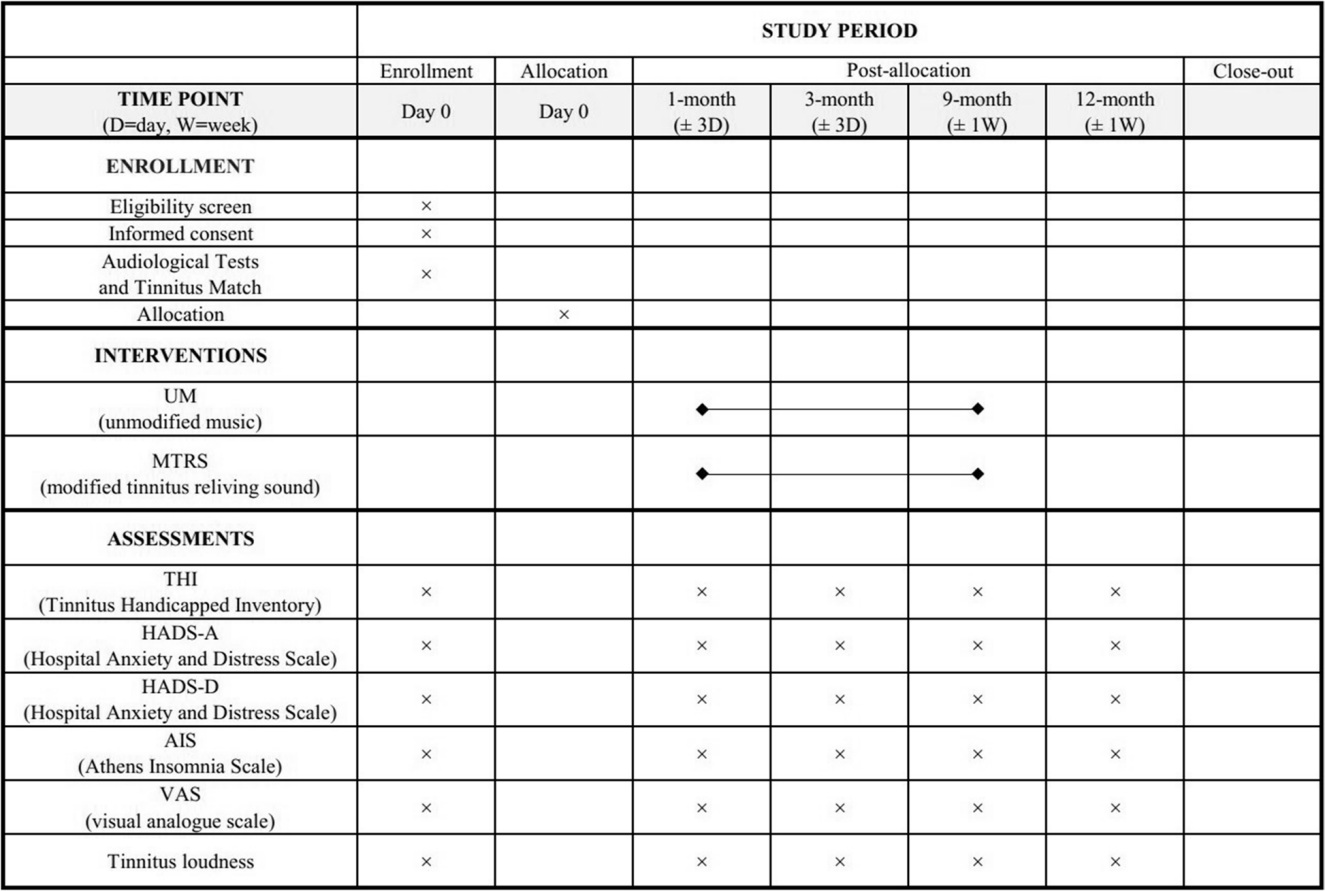
The SPIRIT figure of enrolment, intervention, and assessment

The study flow diagram is shown in Fig. 1, and the SPIRIT figure involving enrollment, intervention, and assessment is presented in Fig. 2.

### Adverse events

Previous studies have shown few advert events of sound therapy. A major possible side effect might be hearing damage caused by the excessive volume of the sound. In this study, we set a strict maximum playback intensity limit to make sure all therapeutic sounds are absolutely less than the discomfort loudness threshold. Besides, our project provides the entire audiology tests for tinnitus match, offering in time pure-tone audiometry (PTA), acoustic immittance (AI) test, and other relevant measurements, along with the independently designed audio processing software/APP and meticulous guidance from our otolaryngologists, whereby the potential injuries could be effectively minimized, and timely intervened.

### Sample size calculation

The study sample size was based on the THI scale (the primary outcome), which is used to assess the severity and treatment effect of tinnitus. Considering an 80% effective rate, 5% significance level, 80% power, 30% dropout, and 1:1 grouping, a total sample of 68 (34 per group) will be required.

### Data analysis

Data analysis will be conducted via statistical software STATA, V. 15.0. The differences in time-varying outcomes between UM and MTRS groups are compared by generalized estimating equations (GEE), which is classified as one sort of generalized linear model (GLM). Subsequently, concomitant variables, including demographic variables, disease data, and tinnitus matching test data at T0, will be weighted and controlled, to further analyze the affecting factors of acoustic therapy for tinnitus. *P* < 0.05 is considered statistically significant and data are presented as means ± standard deviation (SD). All expulsion/withdrawal cases are classified into intention to treat (ITT), in which the miss values would be filled by the method of imputation obtained from the last observation. Meanwhile, per-protocol sets (PP) analysis will be conducted. Finally, compare the ITT and PP results.

### Trial management and quality control

A data monitoring committee (DMC) has been established to store, monitor, and check the authenticity, security, and integrity of the database. All members in the DMC are independent of the study sponsors and declared no competing interests. The DMC will periodically review the accumulated data and communicate the problems of its deliberations to the study team if necessary. The frequency of the interim analyses will be judged by the Chair of the DMC, based on the consultation with the sponsor and principal investigator. We anticipate that there might be 2–3 interim analyses before the final analysis.

Data from the paper surveys will be manually entered into an electronic, password-protected database by trained data entry staff. In case of data discrepancies, data quality inspectors will request data clarification from the researchers, and refer to original files and rules to remove outliers. The CRF was promptly sent to the data statistician in our institution. The data manager should check the data again before data analyses. If there is any problem, the data manager should check the original records, and contact with the researchers. Another trained staff will review and inspect > 20% of all entered survey data to ensure accuracy. The aforementioned original data will be stored on password-protected computers as a backup. All electronic data will be stored on a public platform at http://www.medresman.org, which is sponsored by the China Clinical Trial Registry. The database uses standard techniques to provide security. Access to the database is controlled by user names (principal investigator) and encrypted passwords.

A semi-annual audit will be conducted to protect the integrity of all collected data as part of this trial. The principal investigator will ensure that data collection is conducted and reported according to the protocol. There will be semi-annual auditing trials to ensure the protocol is followed, there are no issues with the informed consent procedure, and record keeping is accurate.

### Confidentiality

All participants enrolled in current trial will be firstly assigned a random number (detailed in the “Randomization and allocation” and “Blinding” part), by which all information collected from participants will be documented. This random number will be the only identifier for participants and there will be no other information that would reveal the participants’ identity. Participants will never be identified by name on consent forms. A list of participants with corresponding identifying random numbers will be made and preserved only by the principal investigator, and this list will be kept on a password-protected computer system, separate from all other data files.

## Discussion

The pathogenesis of tinnitus remains unclear, which is probably related to abnormal variations on the auditory pathway [8]. Tinnitus frequency is often corresponding to the hearing impairment region inside the cochlea [2], thus we invented a strategy to modulate the peaceful music and jointly enhance the upper and lower frequency band adjacent to tinnitus pitch based on the lateral inhibiting effect [27, 28], and the modified sound could be generated and played via our patented software/APP named Fudan Tinnitus Relieving System (FTRS). We plan to verify and compare the clinal efficacy of MTRS to UM in the current RCT. Unmodulated white noise, instrumental music, and natural sounds are often used as controls in clinical trials and they have been generally proven to be less effective than those modified sounds in tinnitus relief [29–31]. Thus, we rationally speculate that MTRS would be effective in reducing negative impacts caused by subjective tinnitus and represent a meaningful advancement in tinnitus intervention.

Anxiety and depression are the common negative emotions concomitant with tinnitus, which in turn aggravate tinnitus suffering, thus forming a vicious cycle [5]. Tinnitus may be transient, intermittent, or persistent in a life-long time, thus education counseling including information support, is crucial for all patients in every phase during a systematic and comprehensive treatment. CBT converts the false and passive perception of tinnitus, reconstructs the positive cognition related to tinnitus, and alleviates the psychological discomforts through various methods, such as relaxation training, mindfulness-based training, and meditation [1]. CBT can observably improve the patients’ quality of life, and alleviate their anxiety and depression [26, 32, 33]. We designed the otologist-leading CBT for tinnitus, that is, figuring out the pathogenesis of tinnitus according to the inspection results and medical history, discussing the possible detriments induced by tinnitus with patients, and providing treatment methods, along with conducting the psychological counseling and behavior training. In this current trial, we plan to integrate customized acoustic therapy with CBT, anticipating to simultaneously mitigate tinnitus suffering and reduce the psychological discomforts (e.g., insomnia, anxiety, and depression) deriving from tinnitus.

A few potential issues related to current RCT should be addressed carefully. This trial has a time-consuming follow-up period, totally having 5 timepoints (when participants are allocated and 1, 3, 9, and 12 months after commencing treatment) for investigators to collect trial data, as a result, there unavoidably will be some participants withdraw from our trial due to different reasons. To reduce the follow-up loss to the maximum, we keep in touch with all participants and timely resolve their encountered problems through multiple ways, such as phone calls, WeChat, and email. Moreover, data input of the assessment scales should avoid manual input errors, by which the electronically input/recording and online submitting will be preferentially adopted, and recorded data will be gathered and exported into statistical analysis software in the background. Also, one assigned investigator will examine the imported data that coming from the CRF (case report form) paper table and dispose of the relevant mistakes or omissions in time.

In conclusion, our trial will concretely and detailly provide the clinical evidence for acoustic therapy by the randomized controlled comparisons between our patented MTRS and UM. Based on preliminary observation and clinical experiences, we hypothesize that the effects of MTRS will be superior to those of UM in terms of the aforementioned outcome measurements and assessment scales. Specifically, we expect that participants in the MTRS group will experience more significantly reduced THI scores, lessened AIS and VAS scores, and better subjective tinnitus relief.

### Study status and recruitment

The planned date of the first enrolment is 15 March 2021. The estimated time required for recruitment is 9 months. The total duration of this study is expected to be 24 months, including statistical analysis, research paper writing, and submitting. This protocol version number is Ver.2.

### Ethics and disseminations

This protocol and the template informed consent forms have been reviewed and approved by the institutional review board (IRB) and the Ethical Committee of Eye & ENT Hospital of Fudan University (Reference Number: 2017047). The research team will make safety and progress reports to the IRB at least annually and within 3 months of study completion. Patients and the public are not involved in the design of this study. The study results will be informed to the public via peer-reviewed journals or academic conferences. The full protocol and dataset will be available from the corresponding author upon reasonable request.
